# Caloric Restriction Alters Postprandial Responses of Essential Brain Metabolites in Young Adult Mice

**DOI:** 10.3389/fnut.2019.00090

**Published:** 2019-06-12

**Authors:** Lucille M. Yanckello, Lyndsay E. A. Young, Jared D. Hoffman, Robert P. Mohney, Mignon A. Keaton, Erin Abner, Ai-Ling Lin

**Affiliations:** ^1^Sanders-Brown Center on Aging, University of Kentucky, Lexington, KY, United States; ^2^Department of Pharmacology and Nutritional Science, University of Kentucky, Lexington, KY, United States; ^3^Department of Molecular and Cellular Biochemistry, University of Kentucky, Lexington, KY, United States; ^4^Metabolon Inc., Durham, NC, United States; ^5^Department of Epidemiology, University of Kentucky, Lexington, KY, United States; ^6^F. Joseph Halcomb III, M. D. Department of Biomedical Engineering, University of Kentucky, Lexington, KY, United States; ^7^Department of Neuroscience, University of Kentucky, Lexington, KY, United States

**Keywords:** caloric restriction, postprandial brain metabolism, metabolomics, neurotransmitters, aging, neurodegeneration, metabolic plasticity

## Abstract

Caloric restriction (CR) has been shown to extend longevity and protect brain function in aging. However, the effects of CR in young adult mice remain largely unexplored. In addition to the fundamental, long-term changes, recent studies demonstrate that CR has a significant impact on transient, postprandial metabolic flexibility and turnover compared to control groups. The goal of this study was to identify the brain metabolic changes at a transient (2 h) and steady (6 h) postprandial state in young mice (5–6 months of age) fed with CR or *ad libitum* (AL; free eating). Using metabolomics profiling, we show that CR mice had significantly higher levels of neurotransmitters (e.g., glutamate, N-acetylglutamate*)*, neuronal integrity markers (e.g., NAA and NAAG), essential fatty acids (e.g., DHA and DPA), and biochemicals associated carnitine metabolism (related to reduced oxidative stress and inflammation) in the cerebral cortex and hippocampus at 2-h. These biochemicals remained at high levels at the 6-h postprandial time-point. The AL mice did not show the similar increases in essential fatty acid and carnitine metabolism until the 6-h time-point, and failed to show increases in neurotransmitters and neuronal integrity markers at any time-point. On the other hand, metabolites related to glucose utilization—glycolysis and pentose phosphate pathway (PPP)—were low in the CR mice throughout the 6-h period and significantly increased at the 6-h time-point in the AL mice. Our findings suggest that CR induces distinct postprandial responses in metabolites that are essential to maintain brain functions. CR mice produced higher levels of essential brain metabolites in a shorter period after a meal and sustained the levels for an extended period, while maintaining a lower level of glucose utilization. These early brain metabolism changes in the CR mice might play a critical role for neuroprotection in aging. Understanding the interplay between dietary intervention and postprandial metabolic responses from an early age may have profound implications for impeding brain aging and reducing risk for neurodegenerative disorders.

## Introduction

Caloric restriction (CR), without malnutrition, has been demonstrated repeatedly to extend lifespan in various species (1). A large body of evidence shows that CR protects brain functions with age, preserves memory in older adults and aging mice, and that CR-treated animals had lower incidence of age-related neurodegenerative disorders, including Alzheimer's disease (2, 3). In particular, the protective mechanism of CR in aging has been suggested to be associated with preservation of neuronal activity, brain metabolic and vascular functions, white matter integrity (WMI), and mitigation of oxidative stress and neuroinflammation (4–10).

The impacts of CR on brain function at an early age are largely unexplored, however. We were interested to know if CR also made significant effects in young adult mice that might be associated with the neuroprotection seen with aging, including metabolites related to neurotransmitters, WMI, glycolysis, and inflammation. Further, the metabolic changes induced by CR may also be postprandial time-dependent. Recent studies demonstrated that individuals with CR had significant differences in transient, postprandial metabolic flexibility, and turnover compared to the control groups (11–13). Therefore, in addition to the fundamental, long-term changes, it will also be important to identify the energy production at different postprandial stages.

In this study, our goal was to identify the brain metabolic changes at transient (2 h) and steady (6 h) postprandial states between young mice fed with CR or *ad libitum* (AL; free eating). We used metabolomics profiling to determine the levels of metabolites of interest. We focused on the brain regions that are highly associated with cognitive functions in rodents, including cerebral cortex and hippocampus. We hypothesized that CR may have significant effects on postprandial brain metabolism in young mice.

## Materials and Methods

### Animals

We obtained male C57BL/6N mice from the National Institute of Aging (NIA) Caloric Restriction Colony with groups of young adult mice (5–6 months of age) (14) fed with either AL or CR diet (*N* = 14 for each group). The 40% CR was administrated to the animals by week 16 and the diet was continued over the lifetime. The vitamin-fortified NIH-31 (NIH-31 fortified) diet fed to CR mice provided 60% of the calories and additional vitamins supplement consumed by *ad libitum* mice. After arriving at our facilities, mice were housed individually (1 mouse per cage) in a specific pathogen-free facility. The CR mice were fed a pellet of the CR diet between 7 a.m. and 9 a.m. everyday. The mice were situated for 3–4 weeks before sending for brain extracts. All experimental procedures were performed according to NIH guidelines and approved by the Institutional Animal Care and Use Committee (IACUC) at the University of Kentucky (UK).

### Metabolomics Profiling

Brain tissue from the cerebral cortex and hippocampus was extracted and homogenized for metabolic profiling. Half of the group (*N* = 7) was subjected under 2-h and the other half under 6-h postprandial brain tissue collection. Brain samples were sent to Metabolon Inc. for biochemical profiling and statistical analysis. Metabolon's standard solvent extraction method was used to prepare the samples, which were then equally split for analysis via liquid chromatography/mass spectrometry (LC/MS) or gas chromatography/mass spectrometry (GC/MS) using their standard protocol (15).

#### Mass Spectrometry Analysis

Non-targeted UPLC-MS/MS and GC-MS analyses were performed at Metabolon, Inc. The UPLC/MS/MS portion of the platform incorporates a Waters Acquity UPLC system and a Thermo-Finnegan LTQ mass spectrometer, including an electrospray ionization (ESI) source and linear ion-trap (LIT) mass analyzer. Aliquots of the vacuum-dried sample were reconstituted, one each in acidic or basic LC-compatible solvents containing 8 or more injection standards at fixed concentrations (to both ensure injection and chromatographic consistency). Extracts were loaded onto columns (Waters UPLC BEH C18-2.1 × 100 mm, 1.7 μm) and gradient-eluted with water and 95% methanol containing 0.1% formic acid (acidic extracts) or 6.5 mM ammonium bicarbonate (basic extracts). The instrument was set to scan 99–1,000 m/z and alternated between MS and MS/MS scans.

Samples destined for analysis by GC-MS were dried under vacuum desiccation for a minimum of 18 h prior to being derivatized using bis(trimethylsilyl)trifluoroacetamide (BSTFA) as described (16). Derivatized samples were separated on a 5% phenyldimethyl silicone column with helium as carrier gas and a temperature ramp from 60° to 340°C within a 17-min period. All samples were analyzed on a Thermo-Finnigan Trace DSQ fast-scanning single-quadrupole MS operated at unit mass resolving power with electron impact ionization and a 50–750 atomic mass unit scan range. The instrument is tuned and calibrated for mass resolution and mass accuracy daily.

#### Compound Identification, Quantification, and Data Curation

Metabolites were identified by automated comparison of the ion features in the experimental samples to a reference library of chemical standard entries that included retention time, molecular weight (m/z), preferred adducts, and in-source fragments as well as associated MS spectra and curated by visual inspection for quality control using software developed at Metabolon (17). Identification of known chemical entities was based on comparison to metabolomic library entries of more than 2,800 commercially-available purified standards. Subsequent QC and curation processes were utilized to ensure accurate, consistent identification and to minimize system artifacts, mis-assignments, and background noise. Library matches for each compound were verified for each sample. Peaks were quantified using area under the curve. Raw area counts for each metabolite in each sample were normalized to correct for variation resulting from instrument inter-day tuning differences by the median value for each run-day, therefore setting the medians to 1.0 for each run. This preserved variation between samples, but allowed metabolites of widely different raw peak areas to be compared on a similar graphical scale.

#### Bioinformatics

The LIMS system encompasses sample accessioning, preparation, instrument analysis and reporting, and advanced data analysis. Additional informatics components include data extraction into a relational database and peak-identification software; proprietary data processing tools for QC and compound identification; and a collection of interpretation and visualization tools for use by data analysts. The hardware and software systems are built on a web-service platform utilizing Microsoft .NET technologies, which run on high-performance application servers and fiber-channel storage arrays in clusters to provide active failover and load balancing.

#### Data Analysis

Log transformations and imputation of missing values with the minimum observed values for each metabolite was performed. Welch's two-tailed *t*-test to was used to identify biochemicals that were significantly different between groups. Levels of statistical significance were reached when *p* < 0.05.

## Results

Table 1 summarizes the category and function of the metabolites that we found significantly different between the CR and AL mice. At the 2-h postprandial time-point, CR mice had significantly higher levels in neurotransmitters, neuronal integrity markers, essential fatty acids, and biochemicals associated with carnitine metabolism compared to the AL mice (Table 2, column 1; CR vs. AL at 2-h). As for neurotransmitters, the CR mice had significantly higher levels of *glutamate, N-acetylglutamate, glycine*, and *serine* (18, 19). *Glutamate* is an excitatory neurotransmitter and associated with cognitive function (20); *glycine* and *serine* (a precursor of *glycine*) are inhibitory neurotransmitters (21). *Glycine* is also anti-inflammatory, cytoprotective, and immunomodulating (20).

**Table 1:** Global list of biochemicals mentioned and their roles.

**Roles**	**Biochemical**	**References**
Neurotransmitters and neuronal integrity markers	Glutamate	(18–22)
	Glycine	
	Serine	
	N-Acetylglutamate (NAG)	
	N-Acetylaspartate (NAA)	
	N-Acetylaspartyl-glutamate (NAAG)	
Essential fatty acids	Dihomolinolenate (20:3n3 or n6)	(23–25)
	Docosapentaenoate (n6 DPA; 22:5n6)	
	Docosapentaenoate (n3 DPA; 22:5n3)	
	Docosahexaenoate (DHA; 22:6n3)	
Carnitine-related metabolites	Carnitine	(26, 27)
	Palmitoylcarnitine	
	Stearoylcarnitine	
	Oleoylcarnitine	
Glucose metabolism and pentose phosphate pathway	Glucose	(28)
	Glucose-6-Phosphate	
	Fructose-6-Phosphate	
	Lactate	
	Alanine	
	Isobar: Ribulose-5-Phosphate, Xyulose-5-phosphate	

**Table 2:** Changes in postprandial brain metabolism between CR and AL mice.

**Pathway**	**Biochemical**	**Column 1**				**Column 2**				**Column 3**			
		**2 h**		**Fold change**		**2 h**	**6 h**	**Fold change**		**6 Hour**		**Fold change**	
		**CR**	**AL**	**CR**	***p*-value**	**AL**	**AL**	**6 h**	***p*-value**	**CR**	**AL**	**CR**	***p*-value**
				**AL**				**2 h**				**AL**	
Neurotransmitters and neuronal metabolites	Glutamate	1.07± 0.092	0.921 ± 0.113	**1.16**	0.002	0.921 ± 0.113	1.01 ± 0.087	1.1	0.056	1.01 ± 0.068	1.01 ± 0.087	1	0.972
	N-acetylglutamate	1.12 ± 0.087	0.911 ± 0.224	**1.23**	0.005	0.911 ± 0.224	0.997 ± 0.048	1.09	0.137	0.977 ± 0.118	0.997 ± 0.048	0.98	0.754
	Glycine	0.987 ± 0.125	0.828 ± 0.219	**1.19**	0.037	0.828 ± 0.219	1.02 ± 0.120	**1.23**	0.021	0.943 ± 0.097	1.02 ± 0.120	0.92	0.444
	Serine	1.05 ± 0.082	0.852 ± 0.201	**1.23**	0.007	0.852 ± 0.201	1.03 ± 0.131	**1.21**	0.017	0.954 ± 0.135	1.03 ± 0.131	0.93	0.368
	N-acetylaspartate (NAA)	1.08 ± 0.036	0.919 ± 0.144	**1.18**	0.001	0.919 ± 0.144	0.995 ± 0.066	1.08	0.092	0.995 ± 0.094	0.995 ± 0.066	1	0.961
	N-acetyl-aspartyl-glutamate (NAAG)	1.34 ± 0.077	0.884 ± 0.288	**1.51**	0.0005	0.884 ± 0.288	1.11 ± 0.355	1.26	0.057	0.974 ± 0.082	1.11 ± 0.355	0.88	0.449
Essential fatty acids	Dihomolinolenate (20:3n3 or n6)	0.900 ± 0.058	0.727 ± 0.156	**1.24**	0.014	0.727 ± 0.156	1.29 ± 0.173	**1.78**	2.76E-07	1.04 ± 0.107	1.29 ± 0.173	**0.81**	0.027
	Docosapentaenoate (n6 DPA; 22:5n6)	0.873 ± 0.076	0.669 ± 0.130	**1.3**	0.014	0.669 ± 0.130	0.993 ± 0.046	**1.48**	0.0008	1.05 ± 0.245	0.993 ± 0.046	1.05	0.864
	Docosapentaenoate (n3 DPA; 22:5n3)	1 ± 0.109	0.779 ± 0.154	**1.28**	0.038	0.779 ± 0.154	1.38 ± 0.275	**1.77**	5.86E-05	0.896 ± 0.176	1.38 ± 0.275	**0.65**	0.001
	Docosahexaenoate (DHA; 22:6n3)	0.985 ± 0.147	0.690 ± 0.134	**1.43**	0.004	0.690 ± 0.134	1.11 ± 0.183	**1.6**	0.0005	0.939 ± 0.212	1.11 ± 0.183	0.85	0.155
Carnitine-related metabolites	Carnitine	1.01 ± 0.164	0.860 ± 0.159	**1.17**	0.045	0.860 ± 0.159	1.06 ± 0.143	**1.23**	0.015	0.925 ± 0.086	1.06 ± 0.143	0.87	0.132
	Palmitoylcarnitine	0.906 ± 0.168	0.629 ± 0.301	**1.44**	0.015	0.629 ± 0.301	1.14 ± 0.266	**1.81**	0.0013	1.05 ± 0.213	1.14 ± 0.266	0.92	0.717
	Stearoylcarnitine	1.09 ± 0.122	0.688 ± 0.333	**1.58**	0.003	0.688 ± 0.333	1.18 ± 0.309	**1.71**	0.002	1.06 ± 0.288	1.18 ± 0.309	0.9	0.595
	Oleoylcarnitine	0.997 ± 0.188	0.640 ± 0.334	**1.56**	0.006	0.640 ± 0.334	1.22 ± 0.364	**1.91**	0.0009	1.13 ± 0.292	1.22 ± 0.364	0.93	0.732

***Column 1** shows the comparison between the CR and AL at 2-h. **Column 2** shows the comparison between AL mice at the 6-h vs. 2-h time point. **Colum 3** shows comparison between the CR and AL at 6-h. Representative data are shown as fold change between groups. The color-coded boxes indicate the change of direction of the metabolites between groups. Data are Mean ± SD. Red and green shaded cells indicate p ≤ 0.05 (red specifies that the mean values are significantly higher for that comparison; green values significantly lower)*.

*N-acetyl-aspartate (NAA)* and *N-acetyl-aspartyl-glutamate (NAAG)* were also found significantly higher in the CR mice at the 2-h time-point. *NAA* and *NAAG* have been used as markers for neuronal integrity as they are most abundant in neurons and are also used as an index of neuron quantity (19); the reduction of these two metabolites have been associated with brain aging and neurodegenerative disorders (22).

CR mice also showed higher levels in dihomolinoleate (20:3n3 or n6), docosapentaenoate (n3 DPA; 22:5n3), docosapentaenoate (n6 DPA; 22:5n6), and docosahexaenoate (DHA; 22:6n3) at the 2-h time-point. These are omega-3, polyunsaturated fatty acids (23). DHA helps with cell membrane structure, assists in normal growth and development, and participates in key pathways of the immune system (24). DPA is often considered the third most prevalent omega-3 fatty acid found in fish oil, following *DHA* and *EPA (eicosapentaenoate)* (25). Carnitine-related metabolites, such as carnitine, palmitoylcarnitine, stearoylcarnitine, and oleoylcarnitine were also higher in the CR mice (26). As carnitine participates in the transport of long-chain fatty acids into the mitochondrial matrix, an increase in these metabolites might indicate facilitation in this transport function and reduced oxidative stress (27).

Interestingly a similar pattern of metabolite increases were not found in the AL mice until the 6-h postprandial time-point (Table 2, column 2; AL, 6-h vs. 2-h). Moreover, some of the metabolites, though increased, did not reached significance, such as *glutamate, N-acetylglutamate, NAA, NAAG*. The results suggest that AL mice may not be as effective in producing these metabolites after a meal, especially those related to improving neuronal integrity.

We further examined the metabolic profile between CR and AL mice at 6-h time-point. At this stage, no significant differences were found in the levels of neurotransmitters, essential fatty acids and glycolytic intermediates between the two groups, except *dihomolinolenate (20:3n3 or n6)* and *docosapentaenoate (n3 DPA; 22:5n3)* (Table 2, column 3; CR vs. AL at 6-h). As these metabolites had an early rise (at 2-h) in the CR group and were followed by the AL group at 6-h, the results indicated that CR mice might have been able to maintain high levels of these metabolites over the 4-h postprandial period.

On the other hand, we found that CR mice had maintained stable levels of glycolytic metabolites over the postprandial period (Table 3). Specifically, *glucose-6-phosphate (G6P)*, fructose*-6-phosphate*, and *lactate* stayed constant in the CR mice, whereas they significantly increased at 6-h in the AL mice; *glucose* was also higher in AL mice at 6-h compared to 2-h, but did not reach significance. A similar pattern was found with *alanine*, an amino acid produced from pyruvate (a product of glycolysis), as well as metabolites associated with pentose phosphate pathway (PPP), including *arabitol* and *xyulose-5-phosphate* and *ribulose-5-phosphate* (28).

**Table 3:** Differences of glycolysis- and pentose phosphate-related metabolites in the young mice.

**Biochemical**	**CR**		**Fold change**		**AL**		**Fold change**	
	**2 h**	**6 h**	**6 h 2 h**	***p*-value**	**2 h**	**6 h**	**6 h 2 h**	***p*-value**
Fructose-6-Phosphate	0.928 ± 0.224	0.993 ± 0.209	1.07	0.597	0.867 ± 0.291	1.16 ± 0.232	**1.34**	0.019
Glucose	0.987 ± 0.213	0.867 ± 0.311	0.88	0.391	0.998 ± 0.303	1.50 ± 0.785	1.5	0.138
Glucose-6-Phosphate	0.965 ± 0.126	0.994 ± 0.222	1.03	0.945	0.860 ± 0.310	1.29 ± 0.279	**1.5**	0.005
Lactate	0.966 ± 0.074	0.904 ± 0.124	0.94	0.353	0.964 ± 0.215	1.14 ± 0.111	**1.18**	0.026
Alanine	0.960 ± 0.122	0.913 ± 0.127	0.95	0.448	0.882 ± 0.143	1.06 ± 0.084	**1.2**	0.009
Isobar: Ribulose-5-Phosphate, Xyulose-5-phosphate	1.14 ± 0.192	0.877 ± 0.158	0.77	0.058	0.927 ± 0.425	1.20 ± 0.344	**1.29**	0.030

*Representative data are shown as fold change between groups. Data are Mean ± SD. The color-coded boxes indicated the direction change of the metabolites between the 2-h and 6-h time points within groups. Red shaded cells indicate p ≤ 0.05 and specify that the mean values are significantly higher for that comparison*.

## Discussion

Caloric restriction is perhaps the most studied intervention that slows down aging and extends longevity since the 1930s (29). CR has been shown to enhance health span and retard aging phenotypes in various systems, including the brain (30). In this study, we further demonstrated that CR also has significant impacts in young animals, especially the distinct postprandial pattern in brain metabolism compared to AL controls. CR mice produced higher levels of many metabolites in a shorter period after a meal, and sustained the levels for an extended period of time. The metabolites included neurotransmitters, neurotrophic factors, essential fatty acids, and carnitine-related metabolism (related to immune function and reduced oxidative stress). The AL mice did not show the similar increases in essential fatty acids and carnitine metabolism until the 6-h time-point, but failed to show increases in neurotransmitters and neuronal integrity markers at any time-point. The findings suggest that CR mice might produce these metabolites more effectively after a meal, especially those related to cognitive functions.

On the other hand, CR mice showed constant lower levels of glucose utilization compared to AL mice. This is consistent with a previous findings using PET-^18^FDG scans that young CR mice had lower glucose uptake in the brain (6). Other studies show that lower glucose uptake was accompanied by higher fatty acids utilization (e.g., ketone bodies), and that this brain metabolic change is preserved with age (7).

Our findings are consistent with Dhahbi et al.'s observations of postprandial responses in metabolic enzyme level induced by CR (11). They showed that CR caused a reduced enzymatic capacity for glycolysis which is consistent with our findings that glycolysis is not up regulated after feeding in CR mice. Further, they found increased activity of glutaminase, an enzyme that converts glutamine to glutamate. This is in line with our observation that CR mice had higher postprandial glutamate levels compared to the AL mice. Collectively, our results are consistent with previous findings that CR altered postprandial patterns in glycolysis and neurotransmitter production.

The findings from the current study led us to speculate that the early changes we saw in the brain metabolites might be associated with the neuroprotective factors seen in aged animals. Indeed, old animals with CR have been shown to have preserved glutamate-glutamine neurotransmission cycling (5), cell structure of white matter (6), cognitive functions (22), and reduced neuroinflammation and oxidative stress (31), and lower incidence for Alzheimer's disease (32, 33). This is also in line with a previous report that early enhancement of cerebral blood flow (CBF) in young mice is associated with CBF preservation in aging mice (8). In other words, the protective effects of CR seen in the aging animals may be manifested as an enhancing factor in young mice. As brain integrity plays a major role in determining lifespan (34), our findings imply the brain metabolic changes observed in the young CR mice may be a critical factor that contributes to the extended lifespan and health span phenomenon that has been repeatedly observed under CR condition.

A limitation of the present study is that we only used male mice; therefore, we were not able to investigate sex effects in the study. Another limitation is that we used a long-lived rodent model. Recent studies have shown that the lifespan response to CR may vary widely in mice from different genetic backgrounds (35). In some cases, CR shortened the lifespan in inbred mice. It will be important in the future to determine if the beneficial effects of CR observed in the young mice in the current study are still warranted in those short-lived inbred mice. Future studies will also need to look into the mechanism of the postprandial turnover in the CR mice.

In conclusion, we demonstrated that CR induces distinct postprandial responses in metabolites that are essential to maintain brain functions, while also maintaining a lower level of glycolysis. Our findings are consistent with literature that CR enhances postprandial metabolic flexibility and turnover. These early changes in CR mice might play a critical role for neuroprotection in aging. Understanding the interplay between dietary intervention and postprandial metabolic responses from an early age may have profound implications for impeding brain aging and reducing the risk for neurodegenerative disorders.

## Ethics Statement

All experimental procedures were performed according to NIH guidelines and approved by the Institutional Animal Care and Use Committee (IACUC) at the University of Kentucky (UK).

## Author Contributions

LMY contributed to the major analysis and interpretation of data for the work. LEAY contributed to the data analysis. RM, MAK, and EA contributed biostatistical support for the metabolomic profiling. A-LL contributed to the major design, analysis and interpretation of data for the work. LMY, JH, and A-LL drafted and revised the work for important intellectual content. LMY, LEAY, JH, RM, MAK, EA, and A-LL approved of the final version and agreed to be accountable for all aspects of the work in ensuring that questions related to the accuracy or integrity of any part of the work are appropriately investigated and resolved.

### Conflict of Interest Statement

The authors declare that the research was conducted in the absence of any commercial or financial relationships that could be construed as a potential conflict of interest.
